# The role of children in transmission of SARS-CoV-2 variants of concern within households: an updated systematic review and meta-analysis, as at 30 June 2022

**DOI:** 10.2807/1560-7917.ES.2023.28.18.2200624

**Published:** 2023-05-04

**Authors:** Yanshan Zhu, Yao Xia, Janessa Pickering, Asha C Bowen, Kirsty R Short

**Affiliations:** 1 School of Chemistry and Molecular Biosciences, The University of Queensland, Brisbane, Australia; 2 Wesfarmer's Centre for Vaccines and Infectious Diseases, Telethon Kids Institute, University of Western Australia, Nedlands, Perth, Australia; 3 Department of Microbiology, State Key Laboratory of Emerging Infectious Diseases, Carol Yu Centre for Infection, School of Clinical Medicine, Li Ka Shing Faculty of Medicine, The University of Hong Kong, Pokfulam, Hong Kong Special Administrative Region, China; 4 Department of Infectious Diseases, Perth Children's Hospital, Nedlands, Perth, Australia; 5 Australian Infectious Diseases Research Centre, The University of Queensland, Brisbane, Australia; * These authors contributed equally to this manuscript

**Keywords:** COVID-19, SARS-CoV-2, variants of concern, children, household transmission

## Abstract

**Background:**

Meta-analyses and single-site studies have established that children are less infectious than adults within a household when positive for ancestral SARS-CoV-2. In addition, children appear less susceptible to infection when exposed to ancestral SARS-CoV-2 within a household. The emergence of SARS-CoV-2 variants of concern (VOC) has been associated with an increased number of paediatric infections worldwide. However, the role of children in the household transmission of VOC, relative to the ancestral virus, remains unclear.

**Aim:**

We aimed to evaluate children's role in household transmission of SARS-CoV-2 VOC.

**Methods:**

We perform a meta-analysis of the role of children in household transmission of both ancestral SARS-CoV-2 and SARS-CoV-2 VOC.

**Results:**

Unlike with the ancestral virus, children infected with VOC spread SARS-CoV-2 to an equivalent number of household contacts as infected adults and were equally as likely to acquire SARS-CoV-2 VOC from an infected family member. Interestingly, the same was observed when unvaccinated children exposed to VOC were compared with unvaccinated adults exposed to VOC.

**Conclusions:**

These data suggest that the emergence of VOC was associated with a fundamental shift in the epidemiology of SARS-CoV-2. It is unlikely that this is solely the result of age-dependent differences in vaccination during the VOC period and may instead reflect virus evolution over the course of the pandemic.

## Introduction

In the first 6 months of the COVID-19 pandemic, numerous household transmission studies suggested that compared with adults, children were less susceptible to severe acute respiratory syndrome coronavirus 2 (SARS-CoV-2) infection and less likely to transmit the virus [1]. These findings were echoed in studies outside of households where the infection rate of SARS-CoV-2 among children younger than 10 years was significantly lower than that of adults [2]. However, since August 2020, the continuous emergence of new variants of SARS-CoV-2 has raised questions as to whether there has been a fundamental shift in the epidemiology of SARS-CoV-2 [3,4].

Globally, there have been at least three peaks corresponding to the circulation of variants of concern (VOC) Alpha (Phylogenetic Assignment of Named *Global Outbreak* (*Pango*) lineage designation B.1.1.7) (or Beta (B.1.351)/Gamma (P.1)), of Delta (B.1.617.2) and of Omicron (B.1.1.529) [3,5]. During these waves, there has been speculation that children have become more susceptible to SARS-CoV-2 infection and more infectious once they contracted the virus. For example, during the Delta wave in Singapore, children (aged 0–11 years) were significantly more likely to transmit and acquire SARS-CoV-2 in a household compared with young adults (18–29 years) [6]. Similarly, during the Omicron wave in the United States (US), the secondary attack rates (SAR) were consistently high across household contact and index age groups, including those aged 0–4 years [7].

Despite these single-site studies, meta-analysis of the role of children in the spread of VOC (relative to the ancestral virus) are generally lacking, with studies often focused on the ancestral virus [8] or not differentiating between data collected during the pre-VOC and VOC-dominant period [9]. Where pre-VOC- and VOC-based studies have been differentiated, data suggest an increased role for children in the household transmission of VOC [8]. However, such analysis remains confounded by the fact that globally, adults have been prioritised for vaccination [10]. Vaccination campaigns for COVID-19 were largely rolled out from December 2020 onwards but with a primary focus on vaccinating individuals 18 years and older. The European Medicines Agency did not approve the vaccination of children 5–11 years of age until November 2021 [11]. Furthermore, paediatric vaccination rates remain consistently lower than those of adults [12]. Vaccination has been shown to reduce the household transmission of SARS-CoV-2 substantially [13]. As a result, it is difficult to ascertain if any observed epidemiological changes in the demographics of viral transmission over time have resulted from a fundamental change in the virus over time or if a potential increase in paediatric infections and transmission is simply indicative of the lower vaccination rate among children.

To assess what effect the SARS-CoV-2 VOC have on children in terms of infectiousness and susceptibility to SARS-CoV-2 infection within a household, we here performed a meta-analysis comparing paediatric SARS-CoV-2 transmission during the pre-VOC- and VOC-dominant period.

## Methods

To use newly published data to further the understanding of the role of children in the household transmission of both ancestral SARS-CoV-2 and SARS-CoV-2 VOC, this systematic review and meta-analysis was performed covering studies published between 25 August 2020 and 30 June 2022.

### Case definitions

We adapted the World Health Organization household transmission investigation protocol for COVID-19 [14]. A household was defined as a group of people (two or more) living in the same residence. Household transmission was defined as two or more positive SARS-CoV-2 cases that occurred in a household within the follow-up period of 28 days after identifying the index. An index case was defined as the first case of laboratory-confirmed COVID-19 in the same household. A secondary case was defined as a known household contact of the index case who tested positive for SARS-CoV-2 during the follow-up period. A household contact was defined as a person who has cohabited with the index case in the same household during the 28 days. In this context, the SAR measured the frequency of secondary infections of COVID-19 among household contacts in a defined period of time, as determined by a positive COVID-19 result. Adults were defined as individuals 18 years and older, while children were defined as individuals younger than 18 years.

### Classification of SARS-CoV-2 variants of concern by study period

Studies were classified as pertaining to the ancestral virus or a VOC based on available genotype data and/or the timing of the study period. Specifically, studies where SARS-CoV-2 genotype was not documented and the index case identification period was before 1 January 2021 were defined as pertaining to data from the period of ancestral virus predominance (pre-VOC period). Studies where data were collected between 1 January 2021 and 30 June 2022 were categorised as pertaining to the VOC period.

### Vaccination status

In investigating the effect of vaccination on transmission, only studies reporting vaccination status of household contacts were included. Vaccinated individuals were defined as those who had received at least one dose of a SARS-CoV-2 vaccine.

### Search strategy and eligibility criteria

**Figure 1:**
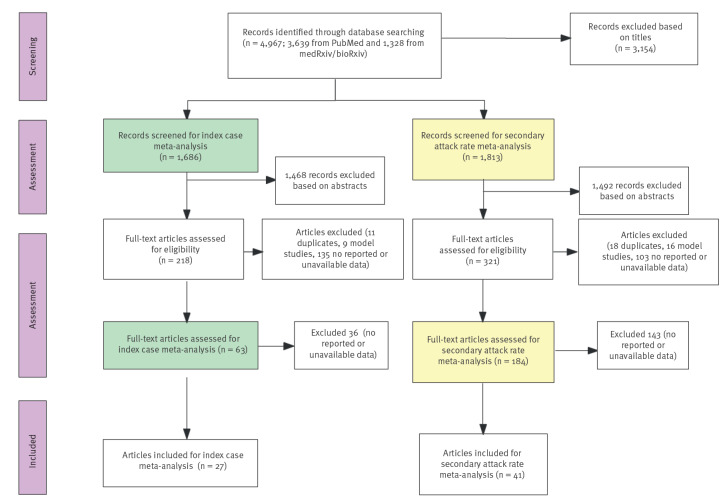
PRISMA flow diagram, systematic review of the role of children in transmission of SARS-CoV-2 variants of concern within households, 25 August 2020–30 June 2022

The literature search was performed in accordance with the Preferred Reporting Items for Systematic Reviews and Meta-Analyses (PRISMA) statement [15]. Our original systematic review had screened literature from 1 December 2019 to 24 August 2020 [1], therefore in this study, publications available between 25 August 2020 and 30 June 2022, were accessed from PubMed, Covid MEDLINE, Embase and Web of Science, using the search term: (“COVID-19” OR “SARS-CoV-2” OR “variant”) AND (“household transmission” OR “family cluster” OR “household contact”) OR (“transmissibility” OR “attack rate”) OR (“vaccination” OR “attack rate”) with no language or location restrictions. Given the role of preprints in timely dissemination of research findings during the COVID-19 pandemic, we also conducted searches of the medRxiv and bioRxiv servers using the search term (“COVID-19” OR “SARS-CoV-2”) AND (“household transmission” OR “secondary attack rate”) for the posted articles. The Strengthening the Reporting of Observational studies in Epidemiology (STROBE) checklists was applied to evaluate the quality of the literature. Investigator YZ developed the initial search strategy, and two researchers (YZ and XY) performed a primary search simultaneously. In cases of difference in opinion, they referred to the selection protocol. If the dispute remained, a third individual (JP or KS) made the final decision. Studies that were duplicate publications, modelling studies, case reports, serological studies and/or reviews were excluded due to a lack of sufficient and/or appropriate data (Figure 1).

### Statistical analysis

We assessed the infectiousness and susceptibility of children and adults to SARS-CoV-2 infection during different time periods by pooling all data deemed eligible by the above selection criteria. Susceptibility to SARS-CoV-2 VOC infection was estimated by pooling the SAR for household contacts. We estimated the relative risk (RR) for SARS-CoV-2 household secondary infection stratified by the age of index cases, the age of household contacts and the vaccination status of household contacts for each study. We then used generalised linear mixed models [16] to estimate pooled RR along with corresponding 95% confidence intervals (CI). As only observational studies were included, we used a random effects model, equalising the weight of the studies to the pooled estimate. Where relevant, we stratified the analysis by pre-specified characteristics including the characteristics of index cases and contacts. A random effects model was used to test subgroup differences. Heterogeneity between studies was evaluated using the I^2^ statistic test. A threshold of I^2^ > 50% indicated statistically significant heterogeneity. All summary analyses and meta-analysis were performed using R studio software (version 3.6.1).

## Results

A total of 4,967 records (3,639 publications and 1,328 preprint articles) were initially identified from the literature search. We rejected 3,154 articles that did not describe SARS-CoV-2 household transmission, leaving 1,813 articles to screen for SAR meta-analysis. Among these studies, 1,492 were excluded based on title and abstract. After further evaluation, the remaining 321 studies were eligible for full-text review. We then screened 1,686 articles for the index case meta-analysis. We identified 48 new studies that satisfied the inclusion criteria. Among these, 27 contained eligible data for the index case meta-analysis, and an additional 41 studies were combined with 11 studies from our previous review [1] to conduct the SAR meta-analysis. Specifically, 29 studies from 16 countries (Australia [17], Bosnia and Herzegovina [18], Brazil [19], Canada [20], China [21-28], Greece [29,30], India [31], Israel [32], Japan [33,34], Malaysia [35], the Netherlands [36], Norway [37], Singapore [38], Spain [39], the United Kingdom (UK) [40,41] and the US [42-45]) were subjected to analysis for SAR in the period defined as pre-VOC. Twenty-three studies involving 12 countries (Denmark [46-49], Germany [50], India [51], Israel [52], Japan [53], the Netherlands [54,55], Norway [56,57], Singapore [6], South Africa [58], Thailand [59], the UK [60,61] and the US [7,9,62-65]) assessed the SAR during the period of VOC dominance. Data from seven studies were only used for the index case meta-analysis [66-72]. All studies newly included in this review are listed in the Table. In general, the quality items of the included studies and data were well reported; we append the full STROBE checklist for the quality of included studies in Supplementary Table S1. Funnel plots for analysis of SAR were drawn and are provided as Supplementary Figure S1.

**Table:** Characteristics of new studies included in the present meta-analysis on the role of children in transmission of SARS-CoV-2 variants of concern within households, 25 August 2020–30 June 2022 (n = 48)

Reference	Location	Diagnosis of COVID-19 case	Study design/period (VOC)	Household size (contacts/index)	Follow-up duration for household contacts	Age of child group (years)	Detection of VOC; vaccination status reported
Sordo et al. 2022^a^ [17]	Australia, New South Wales	Laboratory-confirmed COVID-19 cases	Retrospective observational study; July–October 2020 (pre-VOC)	229 primary cases and 659 close contacts	Secondary cases were defined when a household contact became a confirmed COVID-19 case 2–14 days after the onset date of COVID-19 in the primary case	<18	NA
Musa et al. 2021^a^ [18]	Bosnia and Herzegovina	Laboratory-confirmed cases of SARS-CoV-2 infection	Prospective observational study; 3 August–19 December 2020 (pre-VOC)	360 households and 747 contacts were analysed in this study (747/360).	Households were followed up for a total of 28 days after recruitment	0–11, 12–17	NA
Afonso et al. 2022 [19]	Brazil, Goiânia	Laboratory and clinical epidemiological criteria in line with recommendations of the Ministry of Health of Brazil and the WHO	Cross-sectional study; 15 June–28 October 2020 (pre-VOC)	187 adults were included as index cases. 267 children household contacts were investigated (NA).	Children household contacts were recruited via phone calls and text messages to the adult index case within 10 days of diagnosis	5–9, 10–14, 15–19	NA
Wilkinson et al. 2021 [20]	Canada, Winnipeg Health Region	A confirmed case had laboratory confirmation, with detection of at least one specific gene target by a NAAT assay	Observational study; April 2020 (pre-VOC)	102 primary cases and 279 household contacts	Contacts were followed for the 14-day period	0–19	NA; Y
Li et al. 2021^a^ [26]	China, Wuhan	Laboratory-confirmed cases were individuals with positive detection of SARS-CoV-2 nucleic acid by RT- PCR using respiratory specimens	Retrospective observational study; 2 December 2019–18 April 2020 (pre-VOC)	27,101 households with 57,581 household contacts were identified (24,985 households had only a single primary case)	Household contacts were told to isolate for an additional 14 days at home or designated facilities	0–12, 13–19	NA
Lyngse1 et al. 2021^a^ [46]	Denmark	Positive SARS-CoV-2 laboratory test by RT- PCR	Cross-sectional study: 11 January–7 February 2021 (15% index infected with Alpha)	Index cases in in 5,241 households comprising of 2–6 persons (16,612/8,093)	Those who tested positive in the same household within the following 1–14 days were considered to be secondary cases	1–10, 10–20	Positive samples have been selected for WGS; NA
Lyngse2 et al. 2021 [47]	Denmark	Positive SARS-CoV-2 laboratory test by RT- PCR	Cross-sectional study; December 2021 (Omicron and Delta)	2,225 indexes with the Omicron and 9,712 index cases with the Delta (in total 27,874/11,937)	7-day follow-up period for potential secondary cases	1–10, 10–20	This study relies on variant PCR testing to determine if each primary case was Delta or Omicron; Y
Lyngse3 et al. 2022^a^ [48]	Denmark	Positive SARS-CoV-2 laboratory test by RT- PCR; a primary case was also identified with the Omicron VOC BA.1 or BA.2 by WGS	Cross-sectional study; 20 December 2021–18 January 2022 (Omicron VOC BA.1 and BA.2)	6,419 indexes with the Omicron BA.1 (13,358/6,419) and 2,122 index cases were BA.2 (4,587/2,122).	7-day follow-up period for potential secondary cases	1–10, 10–20	Identified with the Omicron VOC BA.1 or BA.2 by WGS; Y
Lyngse4 et al. 2022 [49]	Denmark	Positive SARS-CoV-2 laboratory test by RT-PCR	Cross-sectional study; 21 June–26 October 2021 (Delta)	Households with 2–6 members, average 53,566 household members per 24,693 indexes	Secondary cases were defined as all cases testing positive within 1–14 days	1–10, 10–20	Delta index was Identified by RT-PCR; Y
Galow et al. 2021^a^ [67]	Germany, Dresden	SARS-CoV-2 PCR-positive	Cross-sectional study; June 2020 (pre-VOC)	139 PCR-confirmed index-case and 238 contacts	NA	<18	NA
Loenenbach et al. 2021^a^ [50]	Germany, Hesse	Positive SARS-CoV-2 laboratory test by PCR	Cross-sectional study; January–February 2021; (Alpha)	The study included 38 households with 92 contact persons	All contact persons were for 14 days followed up daily for symptoms via telephone calls	1–6	NA
Koureas et al. 2021^a^ [29]	Greece, Larissa	Positive SARS-CoV-2 laboratory test by RT- PCR	Cross-sectional observational study; 8 April–4 June 2020; (pre-VOC)	30 households and 223 household contacts	NA	0–12, 13–19	NA
Shah et al. 2021^a^ [70]	India, Gujarat State	Laboratory-confirmed cases	Cross-sectional study, study; March–July 2020 (pre-VOC)	72 paediatric index cases having 287 household contacts were included	Secondary case was defined as individual developing infection within 14 days from last contact with the index case	0–18	NA
Rajmohan et al. 2021 [51]	India, Kerala	Tested for SARS-CoV-2 either by real-time RT PCR or rapid antigen test	Prospective cohort study; 1 January–31 March 2021	101 SARS-CoV-2 index cases and 387 household contacts	Household contacts were followed up for 14 days	0–4, 5–17	NA
Jagdale et al. 2021^a^ [31]	India, Pune City	RT-PCR-positive	Retrospective cohort study conducted in the month of June 2020 (pre-VOC)	119 laboratory-confirmed primary cases and their 741 contacts	The primary contacts that turned RT-PCR positive on throat swab within 14 days of contact (irrespective of symptoms) with the confirmed case were counted in for estimating SAR	<16	NA
Layan et al. 2022 [52]	Israel	Confirmed SARS-CoV-2 infections were defined by a positive PCR test, i.e., with a Cq value lower than 40	Prospective cohort study; 31 December 2020 and 26 April 2021 (Alpha)	210 HCW households with 215 index cases, including four co-index cases and their 687 household contacts (687/215)	At least 10 days of active symptom monitoring	0–12	The study took place when Alpha VOC represented up to 90% of infections in Israel; Y (only individuals ≥ 16 years old were eligible for vaccination)
Ogata et al. 2022^a^ [53]	Japan, Itako	Cases were confirmed using PCR tests with a cycle threshold value of 40	Observational study; December 2020–November 2021 (48% Delta+21% Alpha+31% wt)	The study enrolled 1,257 unvaccinated contacts from 580 households.	NA	0–19	Y
Akaishi et al. 2021 [33]	Japan, Sendai	Positive SARS-CoV-2 laboratory test by real-time RT-PCR	July 2020–March 2021 (pre-VOC)	Household contact group (NA/1,144)	Contact with a COVID-19 patient between 2 days before and 14 days after the onset of symptoms	0–11, 12–17	The study period was well before the replacement of major viral strains spreading in the locality from the original strains to N501Y mutant strains in May 2021
Kuba et al. 2021 [34]	Japan, Okinawa	Confirmed by positive of their clinical specimens (nasopharyngeal swab etc) on SARS-CoV-2 N2 RT-PCR	Observational study; 14 February–31 May 2020; (pre-VOC)	Average (174/78) household members per confirmed index case	The health conditions of the close contacts were followed up for 14 days by PHC staff	0–9, 10–19	NA
Ng1 et al. 2022^a^ [35]	Malaysia, Negeri Sembilan	Confirmed by RT-PCR	Retrospective observational study; 1 February–31 December 2020 (pre-VOC)	The study was conducted among the 185 households (848/185).	The household contacts were placed on strict quarantine at home or at a designated facility for 14 days	0–12, 13–17	The B.1.524 lineages were identified as the predominant circulating variants during the study period; NA
de Gier et al. 2021 [54]	Netherlands	A case was defined as a person with a positive SARS-CoV-2 PCR, loop-mediated isothermal amplification or antigen test	Retrospective cross-sectional study; August–September 2021; (Delta)	The final dataset contained 7,771 contacts of 4,921 index cases (7,771/4,921).	All household contacts were required to quarantine up to 10 days and get tested	12–17	Over 85% Delta variant among sequenced isolates starting from 5 July 2021; Y
Gorgels et al. 2021^a^ [68]	Netherlands	Positive SARS-CoV-2 PCR or antigen test	Retrospective observational study; March 2021–June 2021. alpha variant (B.1.1.7)	97 households and 249 household contacts	Household contacts were followed up for 14 days	4–12	WGS; NA
Soriano-Arandes et al. 2021^a^ [72]	Netherlands	Any individual testing SARS-CoV-2-positive by RT-PCR or by antigen testing in a respiratory specimen	Prospective study; 1 July 2020 and 31 October 2020 (pre-VOC)	NA	NA	0–5, 6–11, 12–16	NA
Verberk et al. 2022^a^ [55]	Netherlands, Belgium and Switzerland	Laboratory-confirmed positive SARS-CoV-2 RT-PCR test result in a household member (index case) and enrolled within 48 h following test result	Prospective cohort study; April 2020 until April 2021	In 276 households with 920 participants (276 index cases and 644 household members) daily (co-primary case)	Self-sampling daily follow-up was continued until 21 days after last symptom onset in any household member	<12,12–18	Households were included before the SARS-CoV-2 vaccination programme was (fully) rolled out and only a small proportion of the population had prior immunity
Reukers et al. 2022 [36]	Netherlands, Utrecht	Laboratory-confirmed SARS-CoV-2 infection was defined as at least one positive PCR on any of the clinical samples	Prospective cohort study; 24 March–6 April 2020 (pre-VOC)	A total of 55 households with 187 household contacts were included (187/55)	All household contacts were tested and subsequently followed up for 4–6 weeks	0–11, 12–17	NA
Jalali et al. 2022^a^ [56]	Norway	Confirmed by PCR test	Retrospective cohort study; December 2021–January 2022 (Delta and Omicron)	In total, 1122 primary cases with confirmed Delta (41%) or Omicron (59%) and 2,169 household contacts (2,169/1,122)	Households of sizes 2–6 individuals, household contacts were monitored for ≤ 10 days after the test date of the primary case	0–16	Virus variant information was based on either PCR variant screening, WGS, or both; Y
Telle et al. 2021 [37]	Norway	Positive PCR results for SARS-CoV-2	Observational study; 1 March 2020 and 1 January 2021 (NA)	The 7,548 families of the index cases comprised 26,991 individuals (26,991/7,548)	Tested positive by PCR within 7 days after the testing date of the index case	0–19	NA
Julin et al. 2021 [57]	Norway, Oslo/Viken	SARS-CoV-2 infection detected by real-time RT-PCR	Prospective longitudinal study; May–June 2020, and September 2020–April 2021 (Alpha and non-VOC)	65 primary cases/households (18 infected with the Alpha variant, one with the Beta variant and 40 with other circulating non-VOC viruses) and their 135 household contacts	The first home visit for inclusion and sampling was termed Day 0, and seven further home visits were followed up for 6 weeks	2–17	WGS
Ng2 et al. 2022^a^ [6]	Singapore	A confirmed COVID-19 case was defined as respiratory specimens positive for SARS-CoV-2 by RT-PCR	Retrospective cohort study; 1 March–31 August 2021 (Delta)	8,470 Delta variant-exposed household close contacts linked to 2,583 index (8,470/2,583)	All identified close contacts underwent legally enforced quarantine for 14 days	0-11, 12-17	All positive cases with RT-PCR Cq < 30 were subjected to WGS for variant identification; Y
Yung et al. 2020 [38]	Singapore	Laboratory confirmation was based on RT-PCR testing of nasopharyngeal swabs	Observational study; March and April 2020 (pre-VOC)	137 households with a total of 223 adults (index patients), 213 paediatric household contacts were included.	Household contacts were quarantined for 14 days from the last day of exposure	0–10, 10–16	NA
Cohen et al. 2022^a^ [58]	South Africa	Positive SARS-CoV-2 laboratory test by RT-PCR	Prospective cohort study; July 2020–August 2021; (wt/Beta/Alpha/Delta)	222 households were included. Average (1,251/222) household members per confirmed index case. We included 180 clusters from 101 households for analysis of HCIR.	Household contacts were prospectively followed active symptom monitoring through the 21-day period	<5, 5–12, 13–18	All positive samples were tested to identify VOC using the AllplexTM SARS-CoV-2 Variants I assay (Seegene Inc., Seoul, Korea); Y
Song et al. 2022^a^ [71]	South Korea	Laboratory SARS-CoV-2-positive cases	Observational study; November–December 2021 (Omicron)	25 households, comprising 55 household members	NA	0–6	NA; Y
Martínez-Baz et al. 2022^a^ [39]	Spain, Navarre	Positive SARS-CoV-2 laboratory test by RT-PCR or antigen test in a specific setting	Prospective cohort study; 11 May–31 December 2020 (pre-VOC)	Average 32,094 household members per 12,829 confirmed index cases	Those who tested positive within the 10 days were counted as cases	<5, 5–14	NA
Posfay-Barbe et al. 2020^a^ [69]	Switzerland	Nasopharyngeal specimens tested for SARS-CoV-2 by RT - PCR	Observational study; 10 March–10 April 2020 (pre-VOC)	39 paediatric index patients and 111 household contacts	The median follow-up of the households was 18 days (IQR: 14–28)	<16	NA
Watanapokasin et al. 2021 [59]	Thailand, Bangkok	Confirmed by RT-PCR	Retrospective study; 1 May–30 June 2021 (Alpha/Delta)	The 30 index cases were associated with 157 exposed household contacts (157/30)	14-day follow-up period for household close contacts	<18	NA; Y
Harris et al. 2021^a^ [60]	United Kingdom	Laboratory-confirmed cases of COVID-19 (HOSTED dataset)	Cross-sectional study; 4 January–28 February 2021 (Alpha)	1,018,842/102,662	14 days observable follow up for all contacts HOSTED dataset	<16	Alpha; Y
Lopez Bernal et al. 2022 [40]	United Kingdom	PCR-positive	Prospective case-ascertained study; January–March 2020 (pre-VOC)	269 primary/co-primary cases resided in 233 homes and 472 household contacts	Trained staff followed up all household contacts of confirmed cases 14 days or more	<18	NA
Miller et al. 2021^a^ [41]	United Kingdom	All SARS-CoV-2 infection laboratory-confirmed by RT- PCR with Cq values ≤39 considered positive	Cross-sectional study; 30 March and 17 November 2020 (pre-VOC)	452 household contacts/181 primary cases	Index cases and their household contacts were followed daily for 14 days to ascertain symptoms and secondary transmission events	0–10, 11–18	NA
Singanayagam et al. 2022 [61]	United Kingdom, Greater London and Bolton	Positive SARS-CoV-2 laboratory test by RT- PCR	Retrospective observational study; 13 September 2020–15 September 2021 (Alpha/Delta/pre-Alpha)	204/138	NA	<18 (aged 5 years or older)	WGS; Y
Chu et al. 2021^a^ [66]	United States, Atlanta	Laboratory tested positive	Retrospective cohort study; 17 July–24 August 2020 (pre-VOC)	224 index patients and 526 household contacts	2 days prior to and up to 10 days after illness onset	7–19	NA
Waltenburg et al. 2022^a^ [9]	United States, California and Colorado	Positive RT-PCR for SARS-CoV-2	Prospective cohort study; January–April 2021 (Alpha)	127 households with a single primary case and 316 household contacts were available for analysis	14-day follow-up period for household close contacts	0–11, 12–17	WGS conducted on at least one nasopharyngeal specimen from participants with a RT-PCR Cq < 35
Donnelly et al. 2022 [62]	United States, California and Colorado	Positive RT-PCR for SARS-CoV-2	Observational study; January–April 2021 (alpha+non-VOC)	127 households with 322 household contacts	The CDC investigators visited households at enrollment (day 0) and at closeout (day 14)	0–17	Nasopharyngeal specimens with an RT-PCR Cq < 35 were selected for WGS; Y
Baker et al. 2022 [7]	United States, four jurisdictions	Positive SARS-CoV-2 nucleic acid amplification test result or antigen test result	Descriptive study and contact tracing; 21 November 2021–3 February 2022 (Omicron)	Enrolled households included 183 index cases and 439 household contacts (439/183)	14-day follow-up	0–4, 5–11, 12–17	Index case with sequence-confirmed Omicron variant; Y
Liu et al, 2021^a^ [63]	United States, Los Angeles, California	Laboratory-confirmed positive SARS-CoV-2 by RT-PCR	Prospective case-ascertained transmission study; December 2020 and February 2021	15 paediatric index cases (<18 years-old) and 50 household contacts	14-day follow-up	<18	NA
Tanaka et al. 2021 [44]	United States, Los Angeles, California	Confirmed by RT-PCR	Retrospective observational study; 17 June–31 December 2020 (pre-VOC)	Households ranged from 2 to 11 members living together (489/105)	Individuals followed up for a median of three visits (IQR: 2–4) over 15 days (IQR: 7–27)	0-11, 12-17	NA; Before vaccination rollout
McLean et al. 2022^a^ [64]	United States, Nashville, Tennessee and Wisconsin	Laboratory-confirmed SARS-CoV-2 infection by RT-PCR	Cross-sectional study; 21 April 2020 to 30 April 2021 (pre-VOC)	404 household contacts/226 primary cases	Index cases and their household contacts were followed daily for 14 days to ascertain symptoms and secondary transmission events	0–4, 5–11, 12–17	NA
Sachdev et al. 2021 [65]	United States, San Francisco	Patients with laboratory-confirmed COVID-19 (positive RT-PCR)	Observational study; 29 January–2 July 2021 (Alpha/Beta/Delta/Gamma)	Among 248 fully vaccinated patients with breakthrough infection, 105 were identified as the index cases (179/105)	NA	<18	WGS; Y
Laws et al. 2021 [42]	United States, Utah and Wisconsin	Positive SARS-CoV-2 laboratory test by RT-PCR or ELISA	Retrospective cohort study; 22 March–25 April 2020 (pre-VOC)	Among 58 households, 188 contacts were enrolled (120 adults; 68 children)	Contacts were assessed daily symptoms prospectively for 14 days and obtained specimens for PCR test and serology testing	<1, 1–4, 5–12, 13–17	NA

CDC: Centers for Disease Control and Prevention; Cq: quantification cycle; HCIR: household cumulative infection risk; HCW: healthcare worker; IQR: interquartile range; NA: not applicable; NAAT: nucleic acid amplification test; PHC: public health centre; SAR: secondary attack rate; SARS-CoV-2: severe acute respiratory syndrome coronavirus 2; VOC: variants of concern; WGS: whole genome sequencing; WHO: World Health Organization; wt: wildtype; Y: Yes.

^a^ 27 studies contained eligible data were included for the index case meta-analysis.

### Infectiousness of children with SARS-CoV-2 within households during the period when the ancestral virus was dominant

**Figure 2:**
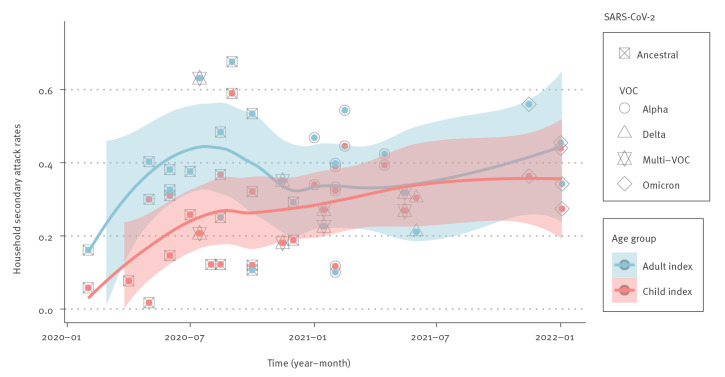
Time pattern for the secondary attack rate from a paediatric vs an adult index case in household SARS-CoV-2 transmission during the respective study periods

SARS-CoV-2: severe acute respiratory syndrome coronavirus 2; VOC: variants of concern.

Fourteen studies were identified that defined the age of the index case and the SAR in the household during the time period in which the ancestral virus was dominant (until 1 January 2021). Another 14 studies were identified that defined the age of the index case and the SAR in the household during the time period in which VOC were dominant. An increasing trend of estimated SAR over time is shown in Figure 2. During the time period when the ancestral virus was dominant (before 1 January 2021), a paediatric index case was associated with a significantly lower SAR compared with an adult index case (RR = 0.61; 95% CI: 0.47–0.80). In contrast, there was no significant difference in SAR (RR = 0.98; 95% CI: 0.85–1.13) between a paediatric index case and an adult index case during the VOC-dominant period. The detailed RR data for the secondary attack rate among household members, when either an adult or a child was identified as the index case, is available in Supplementary Figure S2. The role of children under 12 years in transmitting a VOC within the household was examined by eight observational studies which involved paediatric index cases of different ages with no significant heterogeneity (I^2^ = 19%, p = 0.29). The SAR caused by young paediatric index cases (< 12 years) during the VOC period were higher than SAR attributable to older paediatric index cases (≥ 12 years), in whom we found an estimated 46% significant increase in SAR among household contacts. Supplementary Figure S3 contains a meta-analysis of the SAR among household members, considering both younger and older children as the index case. Taken together, these data suggest that during the period of VOC dominance, children, especially children under 12, were more infectious within households than during the period when the ancestral virus was predominant.

Each time point is based on the midpoint of the study period in each study. Multi-VOC points represent studies with two or more predominant variants. Lines: the smoothed curve of secondary attack rates by month for each group; shaded bands: 95% confidence intervals.

### Secondary attack rate of children in household transmission of SARS-CoV-2 during the circulation of variants of concern

**Figure 3:**
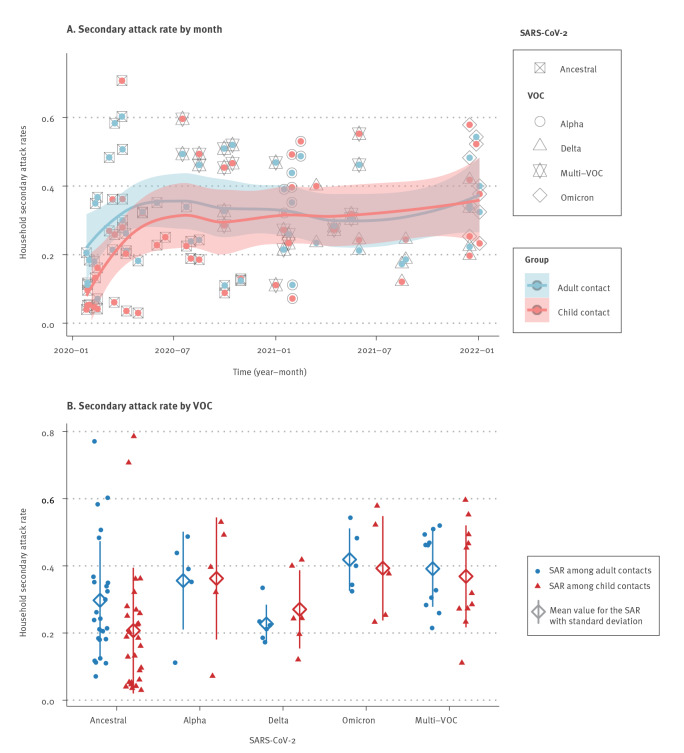
Time pattern for the secondary attack rate among children and adult contacts in household SARS-CoV-2 transmission during the study period and stratified by different VOC, January 2020–January 2022

SARS-CoV-2: severe acute respiratory syndrome coronavirus 2; VOC: variants of concern.

To determine the susceptibility of children to household SARS-CoV-2 infections, the SAR in the household contacts was assessed in 29 pre-VOC studies and 22 VOC studies. The increasing trends of SAR among child and adult contacts in household SARS-CoV-2 transmission was associated with the growing dominance of SARS-CoV-2 VOC since 2021 (Figure 3). The random effects model suggests that children were statistically less likely to acquire ancestral SARS-CoV-2 (SAR = 0.18, 95% CI: 0.12–0.26) than VOC (SAR = 0.31, 95% CI: 0.24–0.38). The raw data used to estimate the pooled SAR of children contacts in household SARS-CoV-2 transmission, stratified by the pre-VOC and VOC period, are available in Supplementary Figure S4. The test of subgroup difference showed there was a statistically significant subgroup effect (p < 0.01). In contrast, before VOC were dominant, the average pooled SAR of adults (SAR = 0.29, 95% CI: 0.23–0.39) was similar to those during the VOC period (SAR = 0.31, 95% CI: 0.26–0.37; p = 0.64). We provide the detailed results on household SAR of adult contacts stratified by the pre-VOC and VOC period in Supplementary Figure S5. As shown in Figure 3B, household SAR among paediatric contacts for VOC were statistically higher than for the ancestral virus (p < 0.001) and equivalent to those among their adult family members (p = 0.93).

Each time point is based on the midpoint of the study period in each study. Multi-VOC points represent studies with two or more predominant variants. Lines in panel A: smoothed curves of secondary attack rates by month for each group; shaded bands: 95% confidence intervals.

**Figure 4:**
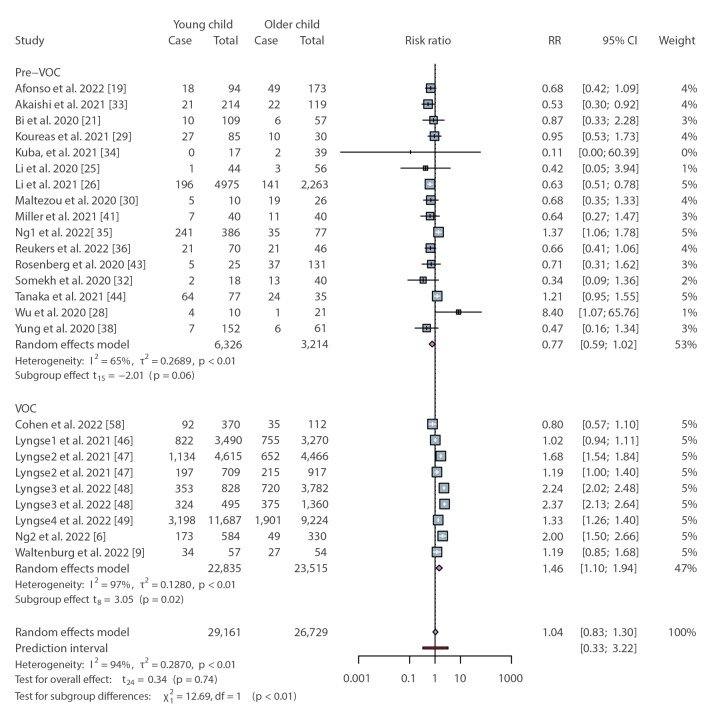
Relative risk for the secondary attack rate of younger and older children contacts in household SARS-CoV-2 transmission stratified by pre-VOC and VOC period

CI: confidence interval; RR: relative risk; SARS-CoV-2: severe acute respiratory syndrome coronavirus 2; VOC: variants of concern.

Although we observed significant heterogeneity between the included studies, in a subset analysis where additional information was provided on the age of the paediatric contacts, younger children (< 12 years) were no more or less susceptible to infection than older children (≥ 12 years) during the pre-VOC period (RR = 0.77; 95% CI: 0.59–1.02) (Figure 4). This is consistent with our prior analysis of the SAR in children and adults during the first year of the COVID-19 pandemic [1]. However, the period of VOC shows a different scenario, in which there was an estimated 46% statistically significant increase in SAR among younger paediatric household contacts compared with older children (RR = 1.46; 95% CI: 1.10–1.94) (Figure 4). In addition, our findings show that compared with older children, the estimated risk of younger children acquiring SARS-CoV-2 was significantly different in the two periods (p < 0.01), although the heterogeneity among the observational studies during the VOC period was high (I^2^ = 97%, p < 0.01).

Cases describe the number of SARS-CoV-2-positive individuals identified in the study.

The above studies were classified as analysing the pre-VOC and VOC periods based on the time when the data were collected. To confirm that the results were the same when specific virus genotyping was performed, we repeated the analysis with a subset of studies where the specific VOC was determined. Consistent with our prior results [1], children were significantly less likely to acquire the ancestral virus in the household compared with adults. We provide extra RR results for the household transmission of SARS-CoV-2 Alpha, Delta, multi-VOC or Omicron, in comparison to the pre-VOC period in Supplementary Figure S6. In contrast, the risk of children being infected with the Alpha, Delta or Omicron variants was not significantly different from exposed adult household contacts when we analysed the RR of SAR among child and adult contacts; the detailed RR results of this analysis can be viewed in Supplementary Figure S6.

**Figure 5:**
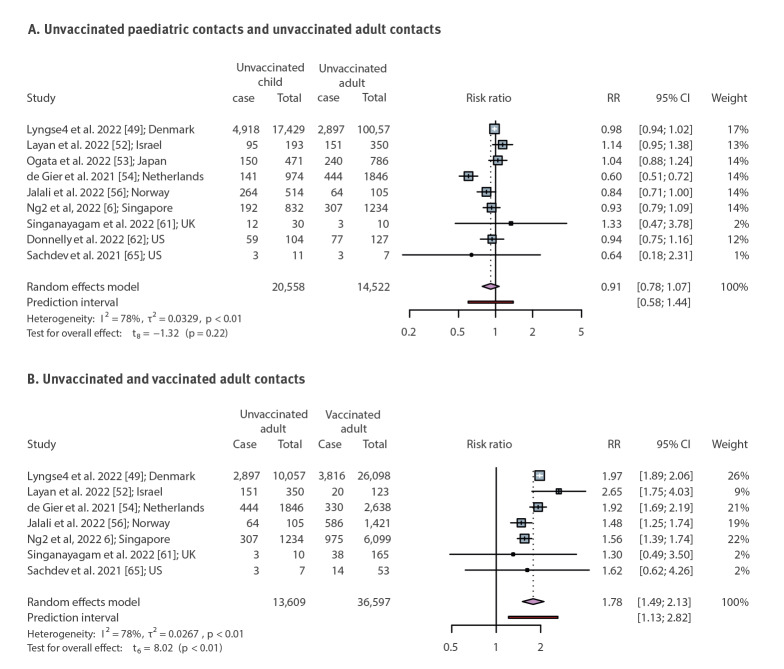
Relative risk for the secondary attack rates of child and adult contacts in household SARS-CoV-2 variant of concern transmission stratified by vaccination status

CI: confidence interval; RR: relative risk; SARS-CoV-2: severe acute respiratory syndrome coronavirus 2; UK: United Kingdom; US: United States.

The above data suggest that children were more infectious (Figure 2) and more susceptible to infection (Figure 3) during the period when VOC were dominant. The household SAR of child contacts stratified by the pre-VOC and VOC period are additionally appended in Supplementary Figure S4. However, this was also the period during which vaccination among adults became widespread. To determine if age-dependent differences in vaccination affected these data, we examined (during the VOC period only) SAR by vaccination status of household contacts regardless of vaccination status or age of the index cases. Only nine studies from Denmark, Israel, Japan, the Netherlands, Norway, Singapore, the UK and the US reported the effectiveness of vaccination against secondary transmission of SARS-CoV-2 within the household. The estimated SAR was higher for unvaccinated adult contacts than vaccinated adults (RR = 1.78; 95% CI: 1.49–2.13) with heterogeneity (I^2^ = 78%, p < 0.01) (Figure 5). These data demonstrate that vaccination can affect SAR within the household. To address this issue in the context of age-dependent differences in vaccination and transmission, we analysed a subset of studies that investigated the SAR in unvaccinated children and unvaccinated adults during the period of VOC dominance. In the absence of vaccination there was no difference in the SAR between adults and children (RR = 0.91; 95% CI: 0.78–1.07) (Figure 5). This is consistent with our prior analysis [1] of the SAR in children and adults. Supplementary Figure S6 contains additional RR results of SAR among child and adult contacts in household transmission of SARS-CoV-2 Alpha, Delta, multi-VOC or Omicron, in comparison to the pre-VOC period.

Cases describe the number of SARS-CoV-2-positive individuals identified in the study. Vaccinated adults were defined as having received at least one dose of vaccine.

## Discussion

In the early stages of the COVID-19 pandemic, children did not appear to play a significant role in the household transmission of SARS-CoV-2 [1]. Data presented here suggest that this has shifted throughout the course of the pandemic and in particular since the emergence of VOC in the community.

The increased role of children in the household transmission of SARS-Cov-2 during the VOC period adds weight to the importance of COVID-19 vaccination programmes in children, including vaccines now available for those younger than 5 years. This remains a contested issue and many parents find it difficult, in light of the typically mild disease children experience when infected with SARS-CoV-2, to make an informed risk–benefit assessment regarding paediatric vaccination [73]. While the slower rollout or even some differing vaccination recommendations [74] for paediatric COVID-19 vaccination have precluded detailed assessments of the precise effect of paediatric vaccination on household SARS-CoV-2 transmission, it is promising that vaccination reduced the risk of infection among cohabitating adults and teenagers [52] and that the probability of person-to-person SARS-CoV-2 transmission between two vaccinated adults and/or teenagers was 4% (compared with the 61% observed between unvaccinated household members) [52]. These data suggest that paediatric COVID-19 vaccination during the VOC period will not only reduce the risk of severe disease in the child but may also play an important role in reducing household transmission of the virus (most probably for a finite period of time after vaccination).

It remains to be determined if the data shown herein can be translated to scenarios outside the home (e.g. SARS-CoV-2 transmission in the school settings). However, even if this should be the case, it is important not to interpret these data as a rationale for re-introducing school closures. In contrast to the early stages of the COVID-19 pandemic, high vaccination rates in adults and the increased availability of paediatric vaccination, combined with a global decline in the severity of COVID-19 cases and improved disease prevention measures (e.g. ventilation, mask use) represent an opportunity for continued face-to-face schooling. However, it is clear that public health decisions such as these need to be derived from data on the current circulating SARS-CoV-2 variants, and not the ancestral virus, to most accurately represent the present situation. Prioritising business as usual for all domains of society and layering public health measures on top of these has become operational in most countries learning to live with COVID-19, and these household transmission data add weight to the importance of this. While children have long been thought to be vectors of high viral transmission, e.g. in the case of influenza virus, SARS-CoV-2 still does not follow this trend. Children are just as likely as adults in the VOC era to transmit SARS-CoV-2, but, in contrast to the seasonal influenza patterns, no more likely than adults [75,76]. In terms of differential infectivity of paediatric age groups, our results also imply that the proportion of transmission that occurs between household members and potentially paediatric age-specific risks could differ in future stages of the pandemic, which is informative for infection prevention within households, as well as schools and childcare.

This study has also provided a new insight into the possible causes of increased VOC transmission among children relative to the ancestral virus. Specifically, our study, in addition to one prior meta-analysis [8], suggests that the role of children in household transmission of SARS-CoV-2 has increased during the VOC-dominant period. It is possible that these data represent differential COVID-19 vaccination rates between children and adults, given the role that vaccination can play in preventing the household transmission of SARS-CoV-2 [13]. However, such a hypothesis would suggest that comparing household transmission among unvaccinated adults and children during the VOC period would show a pattern akin to that of the ancestral virus (i.e. an age-dependence difference in susceptibility to infection and infectiousness within a household). Instead, we have provided valuable evidence that during the VOC period, there were no age-dependent differences in household SARS-CoV-2 transmission among unvaccinated individuals. These data are consistent with a minimal role of differing adult and paediatric vaccination strategies in the changing epidemiology of SARS-CoV-2 during the pandemic. Instead, these data may suggest that the evolution of the virus over time has resulted in an increased role for children in viral transmissions. Indeed, we have recently shown that the ancestral SARS-CoV-2, but not the Omicron variant, is less efficient at replicating in the primary nasal epithelial cells of children, which may have implications for how much virus a child vs an adult shed within the household [77]. However, it does remain possible that the observed shifts in the epidemiology of SARS-CoV-2 over time represent changes in the virus in addition to age-dependent differences in both vaccination and infection. This represents an important area of ongoing research.

This study was subject to several limitations. A high heterogeneity (I^2^) was identified in the data. This is probably attributable to variability in study definitions of index cases and household contacts, frequency and type of testing (we were limited by the information provided in the methods section of each of article), sociodemographic factors, household characteristics (e.g. air ventilation), location of study and local policies (e.g. isolation and quarantine). The often mild nature of SARS-CoV-2 infection in children may have meant that the SAR in transmission studies were underestimated. Alternatively, ongoing exposure from the community (rather than within the household) may have led to overestimating transmission in household settings. Only a limited number of studies were available in the VOC period that documented household SARS-CoV-2 transmission among unvaccinated adults and children, and the definition that those having received one dose of a COVID-19 vaccine were considered vaccinated may have an impact on the findings. Furthermore, the exclusion of prior vaccination but not of prior infection might also have influenced the risk of SARS-CoV-2 infection. Nevertheless, the data provide a comprehensive insight into the shifting role of children in virus transmission over the course of the SARS-CoV-2 pandemic.

## Conclusions

These findings will inform public health strategies and our response to the ongoing emergence of SARS-CoV-2 variants. Specifically, the increased role of children in the household transmission of SARS-CoV-2 during the VOC period adds weight to the importance of COVID-19 vaccination in children. This may also assist parents’ risk–benefit assessment regarding paediatric vaccination, where the benefits can include reducing the household transmission of SARS-CoV-2. In addition, this study has provided a new insight into the possible causes of increased VOC transmission among children relative to the ancestral virus.

## Supplementary Data

1622000 Supplement
