# Author’s correction for Euro Surveill. 2023;28(18)

**DOI:** 10.2807/1560-7917.ES.2023.28.21.230525c

**Published:** 2023-05-25

**Authors:** Eurosurveillance editorial team

**Affiliations:** 1 European Centre for Disease Prevention and Control (ECDC), Stockholm, Sweden

In the article ‘The role of children in transmission of SARS-CoV-2 variants of concern within households: an updated systematic review and meta-analysis, as at 30 June 2022’ by Zhu et al., published on 4 May 2023, the Table listed an incorrect study location for Reference [72]. At the request of the authors, the location was corrected on 22 May 2023 to read “Spain (Catalonia)”.

