# Supplementary material for: The role of children in transmission of SARS-CoV-2 variants of concern within households: an updated systematic review and meta-analysis, as at 30 June 2022
Source: Euro Surveill. 2023 May 4;28(18):2200624. doi: 10.2807/1560-7917.ES.2023.28.18.2200624 (PMC10161681; doi:10.2807/1560-7917.ES.2023.28.18.2200624)
Supplement: Supplement [file 22-00624_SHORT_Supplement.pdf]

Supplementary material

"This supplementary material is hosted by Eurosurveillance as supporting information alongside the article [The role of children in transmission of SARS-CoV-2 variants of concern within households: an updated systematic review and meta-analysis, as of 30 June 2022], on behalf of the authors, who remain responsible for the accuracy and appropriateness of the content. The same standards for ethics, copyright, attributions and permissions as for the article apply. Supplements are not edited by Eurosurveillance and the journal is not responsible for the maintenance of any links or email addresses provided therein.

Contents

TABLE OF CONTENTS	Pages
**Table S1** STROBE checklist of quality items of the included studies.	**2-5**
**Figure S1** Funnel plots for the analysis of the secondary attack rate.	**5**
**Figure S2** Relative risk for the secondary attack rate of household members when an adult or a child was identified as the index case.	**6**
**Figure S3** Relative risk of the secondary attack rate of household members when a young child or an older child was identified as the index case.	**6**
**Figure S4** The secondary attack rate of children contacts in household SARS-CoV-2 transmission stratified by the pre-VOC and VOC period.	**7**
**Figure S5** The secondary attack rate of adult contacts in household SARS-CoV-2 transmission stratified by the pre and VOC period	**8**
**Figure S6** Relative risk of child contact and adult contact in household transmission of SARS-CoV-2 Alpha, Delta, Multi-VOC or Omicron compare with the Pre-VOC period.	**9**

Table S1 STROBE checklist of quality items of the included studies.

Item	No	Recommendation	yes	no	unclear
**Title and abstract**	1	(*a*) Indicate the study's design with a commonly used term in the title or the abstract	6,9,18,19,26, 31,35,37,39, 40-41,44, 51-53, 55,57-59,61,68,70, 72	7,17,20,29,33, 34,36,38,42, 46-50,54,56,60, 62-67,69,71	NA
		(*b*) Provide in the abstract an informative and balanced summary of what was done and what was found	6,7,9,17-20,26,29,31, 33-42, 44,46-59, 61-65,67-70,72	60,66,71	NA
**Introduction**					
Background/rationale	2	Explain the scientific background and rationale for the investigation being reported	6,7,9,17-20,26,29,31, 33-42,44,46-72	NA	NA
Objectives	3	State specific objectives, including any prespecified hypotheses	6,7,9,17-20,26,29,31, 33,35-37,39-42,44,46-72	34,38	NA
**Methods**					
Study design	4	Present key elements of study design early in the paper	6,7,9,17-20,26,29,31, 33-42, 44,46-72	NA	NA

Setting	5	Describe the setting, locations, and relevant dates, including periods of recruitment, exposure, follow-up, and data collection	6,7,9,17-20,26,31,33-42, 44,46-52, 54-59, 60, 62-64, 66, 68-70	29, 53, 61, 65, 67, 71, 72	NA
Participants	6	(*a*) Give the eligibility criteria, and the sources and methods of selection of participants	6,7,9,17-20,26,29,31, 33-42, 44,46-72	NA	NA
Variables	7	Clearly define all outcomes, exposures, predictors, potential confounders, and effect modifiers. Give diagnostic criteria, if applicable	6,7,9,17-20,26,29,31, 33-42, 44,46-72	NA	NA
Data sources/ measurement	8*	For each variable of interest, give sources of data and details of methods of assessment (measurement). Describe comparability of assessment methods if there is more than one group	NA	NA	NA
Bias	9	Describe any efforts to address potential sources of bias	6,7,9,17-18,26,39,41, 44,46-50,52, 55-59,61,62,68, 72	31,33,34,51, 53,54,63-65,69-71	19,20,29,35-38,40,42,60, 66,67
Study size	10	Explain how the study size was arrived at	NA	NA	6,7,9,17-20,26,29,31, 33-42,

					44,46-72
Quantitative variables	11	Explain how quantitative variables were handled in the analyses. If applicable, describe which groupings were chosen and why	6,7,9,17-19,26,29,31, 33-42, 44,46-68,72	71	20,69,70
Statistical methods	12	(*a*) Describe all statistical methods, including those used to control for confounding	6,7,9,17-19 26,29,31, 33-37,39,40,44, 46-52-59,61-65,68-70,72	20,38,71	60,66,67
		(*b*) Describe any methods used to examine subgroups and interactions	6,7,17, 18, 19, 26,29, 33-37,39,40-41,44, 46-50,52-59,60-62,65-66,72	20,31,38,51, 63,64,67-71	NA
		(*c*) Explain how missing data were addressed	6,18,20, 26,29,36,39, 44,52,55-58,61-62,72	7,9,31,33,34, 38,51,53-54,59,60,63, 64,66-71	17,19,35,37, 40-42,46-50,65
		(*d*) If applicable, describe analytical methods taking account of sampling strategy	NA	NA	NA
		(*e*) Describe any sensitivity analyses	6,9,17-19,26, 33, 35-37,39, 40,44, 46-49, 52-59,61-62,72	7,20,31,38,60, 63,64,66-71	29,34,41-42,50,51,65
**Results**					
Participants	13*	(a) Report numbers of	6,7,9,17-	NA	NA

		individuals at each stage of study—eg numbers potentially eligible, examined for eligibility, confirmed eligible, included in the study, completing follow-up, and analysed	20,26,29,31, 33-42, 44,46-72		
		(b) Give reasons for non-participation at each stage	18,20,26, 36, 40,46-49,52,55,57, 61-62,72	38,60,63,66	6,7,9,17,19,29, 31,33-35, 37, 39-42,44,50,51, 53,54,56,58, 59,64,65,67-71
		(c) Consider use of a flow diagram	17-19,33,34, 40-42,52,55,57, 64-65,72	6,7,9,20,26,31,35-39,44,46-51,53,54,56, 58-63,66-71	NA
Descriptive data	14*	(a) Give characteristics of study participants (eg demographic, clinical, social) and information on exposures and potential confounders	6,7,9,17-20,26,29,31, 33-42, 44,46-59, 61-62,64-65,67-72	NA	60,63,66
		(b) Indicate number of participants with missing data for each variable of interest	18,26,36,44, 52, 55,57,58,62, 72	7,9,20,29,31, 33,34,35,37, 38,53,54,59, 60,65-71	6,17,19,36,39-42,46-51,56, 61,63,64
Outcome data	15*	Report numbers of outcome events or summary measures	6,7,9,17-20,26,29,31, 33-42,	NA	NA

			44,46-72		
Main results	16	(*a*) Give unadjusted estimates and, if applicable, confounder-adjusted estimates and their precision (eg, 95% confidence interval). Make clear which confounders were adjusted for and why they were included	6,7,9,17-19,26,29,33-37,39-42, 44,46-63,65-67,72	20,31,38,64, 68-71	NA
		(*b*) Report category boundaries when continuous variables were categorized	6,7,9,17-20,26,29,31, 33-42, 44,46-72	NA	NA
		(*c*) If relevant, consider translating estimates of relative risk into absolute risk for a meaningful time period	NA	NA	NA
Other analyses	17	Report other analyses done—eg analyses of subgroups and interactions, and sensitivity analyses	6,9,17-19,26, 29,33-37,39,44, 46-50, 52-59, 61-62,72	7,20,31,38,40-42,51, 60,63,64,66-71	NA
**Discussion**					
Key results	18	Summarise key results with reference to study objectives	6,7,9,17-20,26,29,31, 33-42, 44,46-72	NA	NA
Limitations	19	Discuss limitations of the study, taking into account	6,7,9,17-20,26,29,33-	31,67	NA

		sources of potential bias or imprecision. Discuss both direction and magnitude of any potential bias	42,44,46-66,68-72		
Interpretation	20	Give a cautious overall interpretation of results considering objectives, limitations, multiplicity of analyses, results from similar studies, and other relevant evidence	6,7,9,17-20,26,29,31, 33-42, 44,46-72	NA	NA
Generalisability	21	Discuss the generalisability (external validity) of the study results	6,7,9,17-20,26,29,31, 33-42, 44,46-72	NA	NA
**Other information**					
Funding	22	Give the source of funding and the role of the funders for the present study and, if applicable, for the original study on which the present article is based	6,9,18,19,26, 29,37,38,39, 41,42,44,46-49,53-59,61-65,67-69,72	7,17,20,31, 33-36,40,50-52,60,66,70, 71	NA

Note: *Give information separately for exposed and unexposed groups. Studies are presented with references order of the manuscript. NA: not applicable.

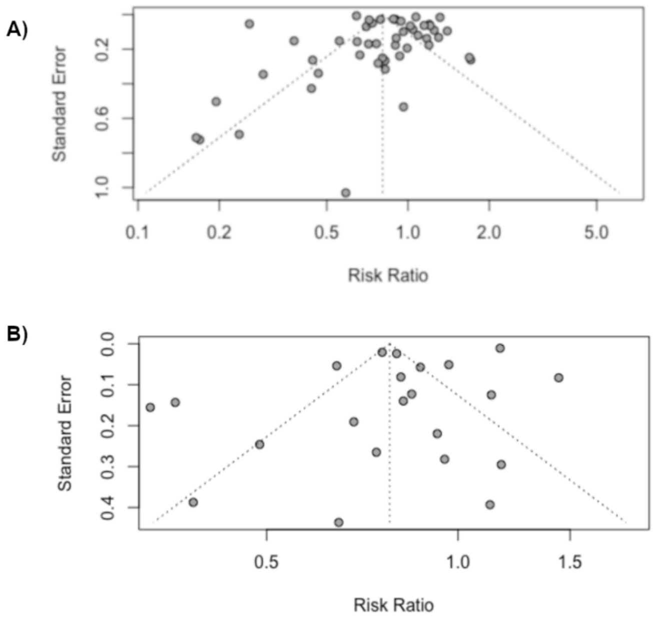

Figure S1. Funnel plots for the analysis of the secondary attack rate (SAR) A) Funnel plots for the analysis of the SAR among children and adult contacts in household SARS-CoV-2 transmission; B) Funnel plots for the SAR of household members when an adult or a child was identified as the index case.

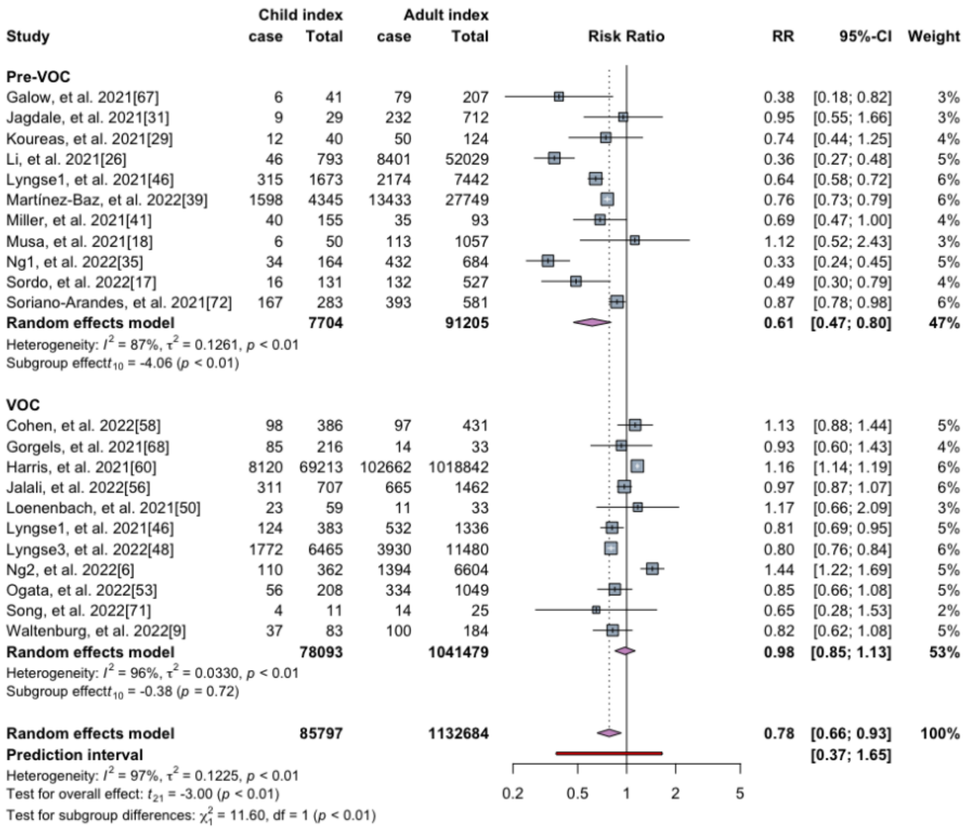

Figure S2. Relative risk for the secondary attack rate of household members when an adult or a child was identified as the index case. Cases describe the number of SARS-CoV-2 secondary infection identified in the study.

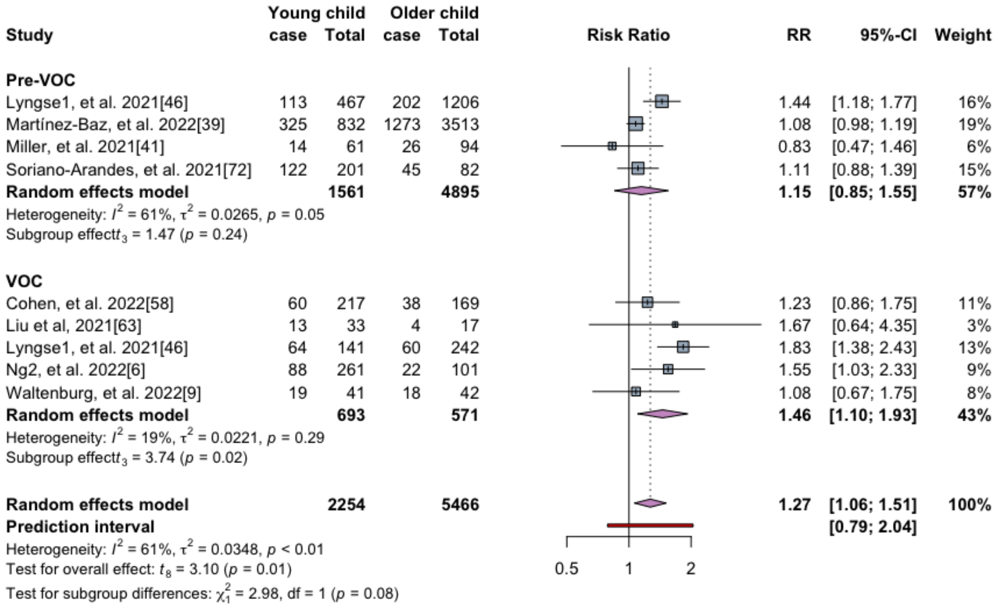

Figure S3. Relative risk of the secondary attack rate of household members when a young child or an older child was identified as the index case. Cases describe the number of SARS-CoV-2 secondary infection identified in the study.

Study; Country	Secondary cases	Total	SAR	95% CI
**Pre-VOC**				
Sordo, et al. 2022[17]; Australia	38	205	0.19	[0.13; 0.25]
Musa, et al. 2021[18]; Bosnia and Herzegovina	17	192	0.09	[0.05; 0.14]
Afonso, et al. 2022[19]; Brazil	67	267	0.25	[0.20; 0.31]
Wilkinson, et al. 2021[20]; Canada	2	67	0.03	[0.00; 0.10]
Bi, et al. 2020[21]; China	16	166	0.10	[0.06; 0.15]
Cheng, et al. 2020[22]; China	1	24	0.04	[0.00; 0.21]
Hua, et al. 2020[23]; China	43	325	0.13	[0.10; 0.17]
Jing, et al. 2020[24]; China	9	172	0.05	[0.02; 0.10]
Li, et al. 2020[25]; China	4	100	0.04	[0.01; 0.10]
Li, et al. 2021[26]; China	337	7238	0.05	[0.04; 0.05]
Wang, et al. 2020[27]; China	13	36	0.36	[0.21; 0.54]
Wu, et al. 2020[28]; China	5	31	0.16	[0.05; 0.34]
Koureas, et al. 2021[29]; Greece	37	115	0.32	[0.24; 0.42]
Maltezou, et al. 2020[30]; Greece	29	41	0.71	[0.54; 0.84]
Jagdale, et al. 2021[31]; India	36	158	0.23	[0.16; 0.30]
Somekh, et al. 2020[32]; Israel	15	58	0.26	[0.15; 0.39]
Kuba, et al. 2021[34]; Japan	2	56	0.04	[0.00; 0.12]
Ng1, et al. 2022[35]; Malaysia	276	463	0.60	[0.55; 0.64]
Reukers, et al. 2022[36]; Netherlands	42	116	0.36	[0.27; 0.46]
Telle, et al. 2021[37]; Norway	2307	12224	0.19	[0.18; 0.20]
Yung, et al. 2020[38]; Singapore	13	213	0.06	[0.03; 0.10]
Martínez-Baz, et al. 2022[39]; Spain	3331	6749	0.49	[0.48; 0.51]
Lopez Bernal, et al. 2022[40]; UK	9	168	0.05	[0.02; 0.10]
Miller, et al. 2021[41]; UK	18	80	0.22	[0.14; 0.33]
Laws, et al. 2021[42]; USA	19	68	0.28	[0.18; 0.40]
Rosenberg et al.,2020[43]; USA	42	156	0.27	[0.20; 0.35]
Tanaka, et al. 2021[44]; USA	88	112	0.79	[0.70; 0.86]
Yousaf, et al. 2020[45]; USA	14	69	0.20	[0.12; 0.32]
**Fixed effect model**		**29669**	**0.23**	**[0.23; 0.24]**
**Random effects model**			**0.18**	**[0.12; 0.26]**
**VOC**				
Lyngse1, et al. 2021[46]; Denmark	1577	6760	0.23	[0.22; 0.24]
Lyngse2, et al. 2021[47]; Denmark	2198	10707	0.21	[0.20; 0.21]
Lyngse3, et al. 2022[48]; Denmark	1772	6465	0.27	[0.26; 0.29]
Lyngse4, et al. 2022[49]; Denmark	5099	20911	0.24	[0.24; 0.25]
Loenenbach, et al. 2021[50]; Germany	9	28	0.32	[0.16; 0.52]
Rajmohan, et al. 2021[51]; India	44	111	0.40	[0.30; 0.49]
Layan, et al. 2022[52]; Israel	95	193	0.49	[0.42; 0.56]
Ogata, et al. 2022[53]; Japan	150	471	0.32	[0.28; 0.36]
de Gier, et al. 2021[54]; Netherlands	231	1911	0.12	[0.11; 0.14]
Jalali, et al. 2022[56]; Norway	326	643	0.51	[0.47; 0.55]
Julin, et al. 2021[57]; Norway	28	60	0.47	[0.34; 0.60]
Ng2, et al. 2022[6]; Singapore	222	914	0.24	[0.22; 0.27]
Cohen, et al. 2022[58]; South Africa	127	482	0.26	[0.22; 0.31]
Watanapokasin, et al. 2021[59]; Thailand	21	38	0.55	[0.38; 0.71]
Harris, et al. 2021[60]; UK	20140	279815	0.07	[0.07; 0.07]
Singanayagam, et al. 2022[61]; UK	12	30	0.40	[0.23; 0.59]
Baker, et al. 2022[7]; USA	70	134	0.52	[0.43; 0.61]
Liu et al, 2021[63]; USA	2	18	0.11	[0.01; 0.35]
Waltenburg, et al. 2022[9]; USA	61	115	0.53	[0.44; 0.62]
Sachdev, et al. 2021[65]; USA	3	11	0.27	[0.06; 0.61]
**Fixed effect model**		**329817**	**0.10**	**[0.10; 0.10]**
**Random effects model**			**0.31**	**[0.24; 0.38]**
**Fixed effect model**		**359486**	**0.11**	**[0.11; 0.11]**
**Random effects model**			**0.23**	**[0.18; 0.29]**

Test for subgroup differences (random effects): $\chi^2_1 = 5.43$, df = 1 (p = 0.02)

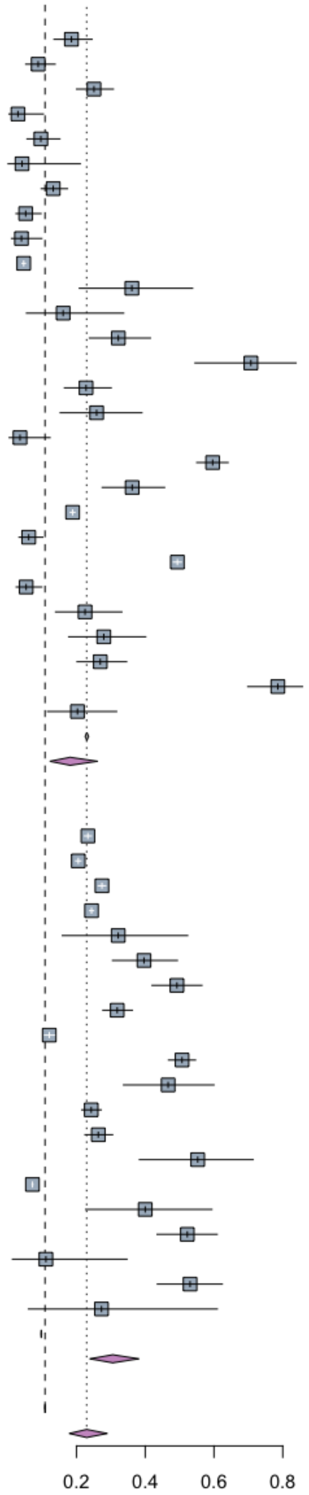

Figure S4. The secondary attack rate of children contacts in household SARS-CoV-2 transmission stratified by the pre-VOC and VOC period. Abbreviation: SAR, secondary attack rate; CI, confidence interval; SARS-CoV-2: severe acute respiratory syndrome coronavirus 2; VOC: variants of concern.

Study; Country	Secondary cases	Total	SAR	95% CI
**Pre-VOC**				
Sordo, et al. 2022[17]; Australia	110	453	0.24	[0.20; 0.29]
Musa, et al. 2021[18]; Bosnia and Herzegovina	95	863	0.11	[0.09; 0.13]
Wilkinson, et al. 2021[20]; Canada	38	209	0.18	[0.13; 0.24]
Bi, et al. 2020[21]; China	61	520	0.12	[0.09; 0.15]
Cheng, et al. 2020[22]; China	9	127	0.07	[0.03; 0.13]
Hua, et al. 2020[23]; China	216	618	0.35	[0.31; 0.39]
Jing, et al. 2020[24]; China	87	774	0.11	[0.09; 0.14]
Li, et al. 2020[25]; China	60	292	0.21	[0.16; 0.26]
Li, et al. 2021[26]; China	7777	43180	0.18	[0.18; 0.18]
Wang, et al. 2020[27]; China	64	299	0.21	[0.17; 0.26]
Wu, et al. 2020[28]; China	43	117	0.37	[0.28; 0.46]
Koureas, et al. 2021[29]; Greece	35	108	0.32	[0.24; 0.42]
Maltezou, et al. 2020[30]; Greece	44	73	0.60	[0.48; 0.72]
Jagdale, et al. 2021[31]; India	205	583	0.35	[0.31; 0.39]
Somekh, et al. 2020[32]; Israel	21	36	0.58	[0.41; 0.74]
Kuba, et al. 2021[34]; Japan	19	90	0.21	[0.13; 0.31]
Ng1, et al. 2022[35]; Malaysia	190	385	0.49	[0.44; 0.54]
Reukers, et al. 2022[36]; Netherlands	36	71	0.51	[0.39; 0.63]
Telle, et al. 2021[37]; Norway	1723	7219	0.24	[0.23; 0.25]
Martínez-Baz, et al. 2022[39]; Spain	11700	25345	0.46	[0.46; 0.47]
Lopez Bernal, et al. 2022[40]; UK	56	304	0.18	[0.14; 0.23]
Miller, et al. 2021[41]; UK	57	168	0.34	[0.27; 0.42]
Laws, et al. 2021[42]; USA	36	120	0.30	[0.22; 0.39]
Rosenberg et al.,2020[43]; USA	88	182	0.48	[0.41; 0.56]
Tanaka, et al. 2021[44]; USA	121	157	0.77	[0.70; 0.83]
Yousaf, et al. 2020[45]; USA	33	126	0.26	[0.19; 0.35]
**Fixed effect model**		**82419**	**0.28**	**[0.28; 0.28]**
**Random effects model**			**0.29**	**[0.23; 0.36]**
**VOC**				
Lyngse1, et al. 2021[46]; Denmark	2556	9852	0.26	[0.25; 0.27]
Lyngse2, et al. 2021[47]; Denmark	4199	17167	0.24	[0.24; 0.25]
Lyngse3, et al. 2022[48]; Denmark	3930	11480	0.34	[0.33; 0.35]
Lyngse4, et al. 2022[49]; Denmark	6713	36155	0.19	[0.18; 0.19]
Loenenbach, et al. 2021[50]; Germany	25	64	0.39	[0.27; 0.52]
Rajmohan, et al. 2021[51]; India	121	276	0.44	[0.38; 0.50]
Layan, et al. 2022[52]; Israel	174	494	0.35	[0.31; 0.40]
Ogata, et al. 2022[53]; Japan	240	786	0.31	[0.27; 0.34]
de Gier, et al. 2021[54]; Netherlands	774	4484	0.17	[0.16; 0.18]
Jalali, et al. 2022[56]; Norway	650	1526	0.43	[0.40; 0.45]
Julin, et al. 2021[57]; Norway	39	75	0.52	[0.40; 0.64]
Ng2, et al. 2022[6]; Singapore	1282	6052	0.21	[0.20; 0.22]
Cohen, et al. 2022[58]; South Africa	68	335	0.20	[0.16; 0.25]
Watanapokasin, et al. 2021[59]; Thailand	55	119	0.46	[0.37; 0.56]
Harris, et al. 2021[60]; UK	82522	739027	0.11	[0.11; 0.11]
Singanayagam, et al. 2022[61]; UK	41	175	0.23	[0.17; 0.30]
Baker, et al. 2022[7]; USA	157	289	0.54	[0.48; 0.60]
Liu et al, 2021[63]; USA	15	32	0.47	[0.29; 0.65]
Sachdev, et al. 2021[65]; USA	17	60	0.28	[0.17; 0.41]
Waltenburg, et al. 2022[9]; USA	76	156	0.49	[0.41; 0.57]
**Fixed effect model**		**828604**	**0.13**	**[0.12; 0.13]**
**Random effects model**			**0.31**	**[0.26; 0.37]**
**Fixed effect model**		**911023**	**0.14**	**[0.14; 0.14]**
**Random effects model**			**0.30**	**[0.26; 0.35]**

Test for subgroup differences (random effects): $\chi^2_1 = 0.22$, df = 1 ($p = 0.64$)

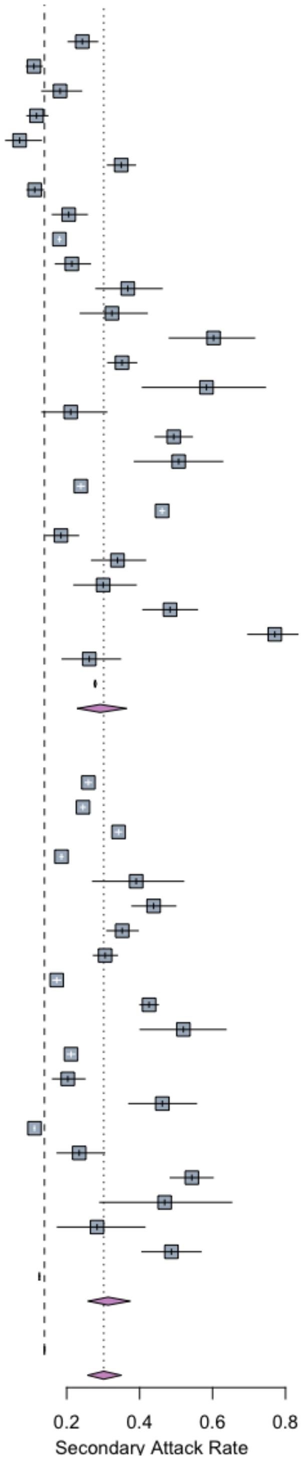

Figure S5. The secondary attack rate of adult contacts in household SARS-CoV-2 transmission stratified by the pre and VOC period. Abbreviation: SAR, secondary attack rate; CI, confidence interval; SARS-CoV-2: severe acute respiratory syndrome coronavirus 2; VOC: variants of concern.

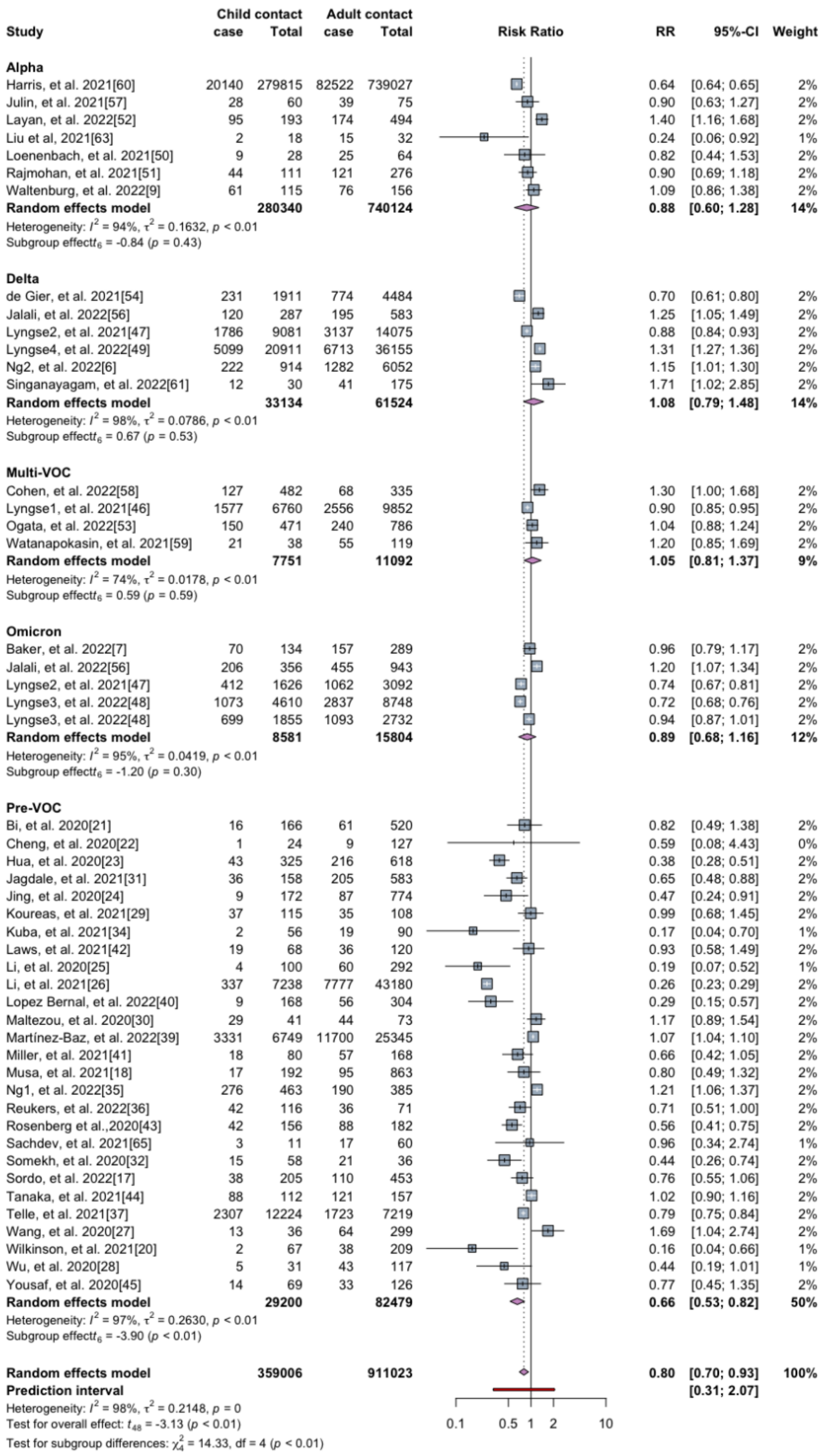

Figure S6. Relative risk of child contact and adult contact in household transmission of SARS-CoV-2 Alpha, Delta, Multi-VOC or Omicron compare with the Pre-VOC period. Cases describe the number of SARS-CoV-2 secondary infections identified in the study. Abbreviation: CI, confidence interval; RR: relative risk; VOC: variants of concern.
